# Genetically Improved Yeast Strains with Lower Ethanol Yield for the Wine Industry Generated Through a Two-Round Breeding Program

**DOI:** 10.3390/jof11020137

**Published:** 2025-02-11

**Authors:** Eduardo I. Kessi-Pérez, Melissa Gómez, William Farías, Verónica García, María Angélica Ganga, Amparo Querol, Claudio Martínez

**Affiliations:** 1 Centro de Estudios en Ciencia y Tecnología de Alimentos (CECTA), Universidad de Santiago de Chile (USACH), Santiago 9170022, Chile; eduardo.kessi@usach.cl (E.I.K.-P.); melissa.gomez.r@usach.cl (M.G.); veronica.garcia@usach.cl (V.G.); 2 Departamento de Ciencia y Tecnología de los Alimentos, Universidad de Santiago de Chile (USACH), Santiago 9170002, Chile; angelica.ganga@usach.cl; 3 Laboratorio de Fisiología y Genética Marina (FIGEMA), Centro de Estudios Avanzados en Zonas Áridas (CEAZA), Coquimbo 1781421, Chile; william.farias.munoz@gmail.com; 4 Departamento de Biotecnología de los Alimentos, Instituto de Agroquímica y Tecnología de los Alimentos (IATA), CSIC, 46980 Valencia, Spain; aquerol@iata.csic.es

**Keywords:** yeast, ethanol yield, genetic improvement, breeding program, microsatellites, wine, wine industry

## Abstract

*Saccharomyces cerevisiae* is a species of industrial significance in the production of alcoholic beverages; it is the main species responsible for the fermentation of grape must. One of the main current problems in the wine industry is high alcohol levels caused by climate change. Pre- and post-fermentation strategies are used to reduce the alcohol content in wines; however, they are inefficient, affect organoleptic properties, face legal restrictions, and/or increase production costs, which has motivated efforts to obtain microbiological solutions. In the present work, we carried out a two-round breeding program to obtain improved yeast strains with lower ethanol yield. The trait under study showed high heritability (0.619), and we were able to lower the ethanol yield by 10.7% in just one generation. We finally obtained a population composed of 132 strains, of which 6 were used to produce wine from natural grape musts on a pilot scale, highlighting improved strains “C2-1B4” and “C7-1B7” as those that showed the best results (alcohol levels between 0.3 and 1.5% ABV less than expected). Further studies are required to understand the connection between initial sugar concentration and ethanol yield, as well as the genetic variants underlying this phenotype.

## 1. Introduction

*Saccharomyces cerevisiae* is a yeast species of industrial significance given its role in the production of several alcoholic beverages; it is the main species responsible for the fermentation of grape must in wine production [1]. In general terms, wine fermentation is a complex microbiological process where *S. cerevisiae* overcomes its competitors by transforming sugar (glucose and fructose) into ethanol [2]. However, although the main contribution of yeast is the alcohol level, it also contributes flavors and aromas to the produced wine [3,4].

One of the main current problems in the wine industry is high alcohol levels in wine due to high concentrations of sugar in the grapes at harvest time (i.e., grape musts with levels of 25 °Bx or more), a recurring observation in recent years. This situation has been correlated with global climate changes, which have caused a mismatch between the appropriate harvest time according to oenological parameters and the sugar concentration in the grapes. If the climate is warmer than ideal, vines will undergo phenolic development more quickly, resulting in earlier sugar ripening and a loss of acidity through respiration as flavors develop. The result is unbalanced or “flabby” wines (wines with high alcohol content and little acidity retained for freshness) [5]. In the last 30 years, the average alcohol concentration in wines has increased by an average of 2% ABV (alcohol by volume) [6].

This situation has made the alcoholic content in wines a relevant issue for the wine industry today, forcing the sector to implement strategies that allow for reductions in alcohol content without harming those attributes that make it possible to achieve highly competitive wines. High alcohol content in wines affects competitiveness in different dimensions: Commercially, wines with high levels of alcohol increase the tax burden; sensorially, flavors and aromas are masked, and, to a lesser extent, the perception of sweetness and acidity is reduced; productively, the winemaking process is inhibited, generating stagnant or slow fermentations [7,8].

Currently, pre- and post-fermentation strategies are used to reduce the alcohol content in wines [9,10,11,12]. For example, pre-fermentation actions include the dilution of the must with others with lower sugar content, the addition of enzymes that degrade sugars, the addition of a fraction of water, crop management, and early harvesting of the fruit (in detriment of oenological maturity). On the other hand, post-fermentation actions involve directly intervening in the wine through methods that reduce alcohol content, with reverse osmosis processes being widely used. However, these strategies are inefficient, affect the organoleptic properties of the wine, face legal restrictions, and/or increase production costs.

In recent years, efforts have been made to generate microbiological solutions for this problem. The simplest strategy is to search for strains in nature with low ethanol yields, a strategy used for the selection of a large portion of the globally marketed wine yeast strains [13], or even to use mixed cultures with yeasts less efficient for alcohol production [14]. One example of the latter strategy is the use of commercial non-conventional yeasts like *Metschnikowia pulcherrima* (e.g., the Maurivin AWRI Obsession strain) in co-culture with *S. cerevisiae* strains [15]. However, this type of strategy has faced problems such as increased fermentation times, complexity and uncertainty, and with the different techniques developed in recent decades for yeast genetic modification and with the accumulated knowledge on the genetic basis of yeasts with different fermentation-related phenotypes, more rational strategies could be used.

One of the main efforts seen recently is the generation of *S. cerevisiae* strains with a lower yield in ethanol production through genetic engineering [14,16]. However, this strategy has faced criticism due to the public and commercial rejection of genetically modified organisms (GMOs), making it unviable for many important markets (most notably Europe) [17]. Therefore, the alternative option is to perform genetic modification without using genetic engineering techniques. One possible strategy is to use adaptive laboratory evolution (ALE) to obtain *S. cerevisiae* strains with the desired phenotype, as was performed in the generation of the commercial strain IONYSwf™ (Lallemand), which exhibits reduced alcohol production, but only in a single variety of wine (Shiraz), leaving a large market without a solution [6].

An alternative genetic modification strategy is the combination of whole genomes by hybridization, which does not face the same legal restrictions as GMOs. This strategy has been used to generate new strains for the wine industry, performing both intraspecific and interspecific crossings [18,19,20,21,22], for example the Velluto Evolution^TM^ (Lallemand) strain, a hybrid between a commercial *S. cerevisiae* strain and *Saccharomyces uvarum* [23]. However, in most cases, this approach has been carried out using only commercial strains, restricting the genetic variability found in the parental strains. One way to take better advantage of the natural genetic variability present in the species is to carry out breeding programs (also known as genetic improvement programs) using a very diverse initial population, similar to those commonly used to obtain new varieties of plants and animals, but unfortunately, this strategy has been rarely used in yeast [13], and, to our knowledge, it has not been used to generate wine yeast strains with reduced ethanol yield.

In the present study, we carried out a two-round breeding program starting from an F1 generation previously generated from a group of 70 strains isolated from winemaking environments mainly in Chile and Argentina (i.e., the original F0 population) [13]. Two rounds of breeding were carried out, the first to reduce the ethanol yield (generating an F2 population), and then the second to reduce residual glucose (generating an F3 population). It was found that both traits under study have high heritability, allowing us to obtain strains that produce less alcohol while leaving minimal residual sugars. Among these improved strains, strains “C2-1B4” and “C7-1B7” in particular showed marked reductions in their ethanol production efficiencies without losing fermentative performance. When using these improved strains to produce wine from natural grape musts under winery conditions, strains “C2-1B4” and “C7-1B7” were able to produce wines with alcohol levels between 0.3 and 1.5% ABV less than expected based on the sugar levels of the grape musts used, for white and red wine (respectively); on the other hand, sensory analyses confirmed that these wines were equivalent to those of wines produced with industrial yeasts in terms of organoleptic properties. Therefore, the characteristics of improved strains “C2-1B4” and “C7-1B7” may be of commercial interest to the wine industry.

## 2. Materials and Methods

### 2.1. Yeast Populations and Breeding Program Design

To carry out the breeding program, we started from an F1 generation, composed of 195 *S. cerevisiae* intraspecific hybrid strains (hereinafter, “intraspecific hybrids” will be simply referred to as “hybrids”) previously generated by random crosses between individuals of an F0 population (composed of 70 strains isolated from winemaking environments mainly in Chile and Argentina) [13] (Table S1). It was previously verified that all strains in the F0 population were of the *S. cerevisiae* species, so all strains in the F1, F2 and F3 populations are also *S. cerevisiae*. Then, two rounds of breeding were carried out: The first was conducted to reduce ethanol yield (generating an F2 population), and the second was conducted to reduce residual glucose (generating an F3 population). Sporulation and hybrid formation by pairwise crosses were performed as previously described [24,25], performing spore-to-spore matings using a dissection microscope SporePlay+ (Singer Instruments, Watchet, UK). Molecular identification of true hybrids was performed using between 1 and 4 microsatellites (SSRs) for each cross from an initial pool of 33 SSRs: YBL084C, YBR058C, YBR240C, YDL132W, YDR160W, YGL035C, YGL184C, YKR014C, YLL049W, YLR013W, YML091C, YOR267C, YPL009C, C3, C4, C5, C6, C8, C9, C11, ScAAT1, ScAAT5. YKL172W, YKR072C, SSR77503chr1Sc, SSR68291chr2Sc, SSR72355chr2Sc, SSR464026chr2Sc, SSR541452chr2Sc, SSR390431chr4Sc, SSR142898chr5Sc, SSR186218chr6Sc and SSR 431413chr7Sc [13,26,27,28,29].

The genetic-additive variance (V_A_) of the traits was estimated through intraclass correlations, using the restricted maximum likelihood (REML) method [30]. The ASReml v.4.0 program was used to estimate heritability from a pedigree, using mixed linear models of analysis of variance, according to the general model [31]:

(1) Y=Xb+Za+e

where Y is a vector of observations of all individuals, b is the vector of fixed effects, a is the vector of additive genetic effects (random effects in the model) and e represents the residual effects caused by non-additive genetic and uncontrolled environmental effects. X and Z are the corresponding incidence matrices. Heritability (h^2^) was calculated as the ratio of the additive genetic variance to the phenotypic or total variance [32,33]. The statistical significance of heritability was estimated using the log-likelihood ratio test (log-LR test) [34].

On the other hand, the expected response to selection (G) was estimated as follows:

(2) $$G=h^{2}\cdot i\cdot \sqrt{V_{P}}$$

where h^2^ corresponds to the estimated heritability, i corresponds to the selection intensity and VP corresponds to the phenotypic variance.

Finally, the percentage of response to selection (%G) was estimated as follows:

(3) $$\%G=\frac{G}{\bar{X}}\cdot 100$$

where G corresponds to the expected response to selection and $\bar{X}$ is the mean of the variable of interest.

Statistical analyses of population traits consisted of one-way ANOVA using Holm–Šídák’s multiple comparisons tests, which were performed using Graph Pad Prism 7.04 software.

### 2.2. Microscale Fermentations with Synthetic Must

Microscale fermentations of F2 and F3 populations and the commercial strain Lalvin EC1118^TM^ (Lallemand Inc., Montréal, QC, Canada) were carried out as previously described [35,36] with some modifications. Each strain was fermented in triplicate in a commonly used synthetic must [37,38], which had 300 mg/L of yeast assimilable nitrogen (YAN), with a high concentration of sugars (250 g/L total, 125 g/L glucose, and 125 g/L fructose, corresponding to approximately 24.8 °Bx). For each experiment, strains were initially grown under agitation in 5 mL of synthetic must overnight at 25 °C. Next, 1 × 10^6^ cells/mL were inoculated into 12 mL of synthetic must (in 15 mL conical tubes) and incubated at 25 °C without agitation. Fermentations were weighed daily to calculate the CO_2_ loss until the CO_2_ loss was less than 10% of the accumulated CO_2_ lost (at this point, the fermentation was considered finished). At the end of the fermentation, the replicas were centrifuged at 9000× *g* for 10 min and the supernatant was collected. Aliquots of the fermented synthetic must were injected into Shimadzu Prominence HPLC equipment (Shimadzu, Kyoto, Japan) using a Bio-Rad HPX-87H column (Hercules, CA, USA) [39], to obtain the values for residual glucose and fructose, and for the production of acetic acid, glycerol, and ethanol.

### 2.3. SO_2_ Resistance Test

An evaluation of the SO_2_ resistance of 17 selected improved strains and the commercial strain Lalvin EC1118^TM^ (Lallemand Inc., Montréal, QC, Canada) was carried out in triplicate by monitoring the OD_600_ of the cells in 30 min intervals on a Tecan Sunrise microplate reader (Tecan, Männedorf, Switzerland), and calculating the area under the growth curve (AUC) of each strain using the Graph Pad Prism 7.04 software, as previously described [40]. Each strain was grown for 62 h at 30 °C in synthetic must, supplemented with five different SO_2_ concentrations (20, 40, 60, 80, and 100 ppm), and for each strain, the percentage of the AUC relative to the absence of SO_2_ in each condition was calculated.

### 2.4. Pilot-Scale Fermentations with Natural Grape Must

Fermentations with the natural grape must of six selected improved strains were carried out in triplicate in 10 L tanks using two types of grapes must: Sauvignon blanc (22.8 °Bx, initial density 1101 kg/m^3^) and Carmenere (24.4 °Bx, initial density 1103 kg/m^3^). In each case, the grape must was treated with SO_2_ (and for the Carmenere must, total acidity was corrected using tartaric acid), and then each tank was inoculated at a concentration of 1 × 10^6^ cells/mL of cells pre-grown on a 2 L Biostat B bioreactor (Sartorius, Göttingen, Germany), using a working volume of 1 L, as previously described [41], for 48 h at 28 °C. Fermentation was monitored by the daily measurement of density and temperature (14 °C for Sauvignon blanc and 18 °C for Carmenere), and supplementations with ammonium phosphate and other nutrients were carried out at specific densities for each must. Implantation analyses of the different strains were carried out by isolating 11 yeast colonies once each fermentation was completed and identifying them by RFLP-mtDNA as previously described [42,43,44]. Fermentations with Carmenere must also included a step of malolactic fermentation. At the end of fermentation, after verifying that each fermentation occurred correctly, the replicas were pooled into a single stock, and the wines were racked, physically and chemically stabilized, bottled without filtration, and stored at 4 °C. The final chemical wine parameters (reducing sugars and alcoholic degree) were analyzed in the same way they are routinely analyzed by the wine industry, that is, by commissioning the chemical analysis to a certified independent company (Vinotec Chile S.A., Santiago, Chile). The genetic stability of the final selected strains was evaluated by mitochondrial DNA (mtDNA) restriction fragment length polymorphism (RFLP) [44] and pulsed-field gel electrophoresis (PFGE) [45], as previously described [42,43].

### 2.5. Sensory Analysis

An independent panel made up of nine expert judges (oenologists who regularly carry out wine tastings) carried out a sensory analysis of the wines produced with the improved strains. Wines were evaluated after 7 months (white wines) and 4 months (red wines) of bottle aging, and two types of sensory tests were carried out, one affective (or of preference) and another descriptive, following previously published guidelines [46]. The affective test, which is recommended to evaluate more than two samples at a time, measured the degree of satisfaction through ratings using a 5-point hedonic scale from 1 (“I dislike it”) to 5 (“I like it a lot”), while in the descriptive test, several attributes were measured in terms of aroma (mineral, general aroma, spice, floral, strawberry, ripe fruit, vegetable, and reduced) and flavor (bitterness, acidity, alcohol, and sweetness, in addition to structure and persistence in the case of red wines) [47], using a perception intensity scale from 0 (“absence”) to 5 (“extreme”) points. To minimize carry-over effects, panelists were asked to follow a sip and spit protocol, rinsing their mouths with mineral water between samples [48]. Statistical analysis of sensory parameters consisted of independent ANOVA and LSD Fisher tests, which were performed using R 4.1.2 software [49].

## 3. Results

### 3.1. Two-Round Breeding Program to Obtain Strains with Less Ethanol Yield

To obtain yeast strains with lower ethanol yields, we carried out a breeding program starting from a first filial generation (F1 population) composed of 195 hybrid individuals previously generated and characterized (21) (Table S1). The F1 population showed a mean value for this trait of 0.506 g ethanol/g sugar, with a minimum of 0.365 g ethanol/g sugar and a maximum of 0.568 g ethanol/g sugar, and a heritability value of 0.619 (Table 1 and Table 2 and Figure S1).

In total, 35 strains (17.9%) of the F1 population were selected as parental strains to produce a second filial generation (F2 population), where 22 families of full siblings and 13 families of half siblings were represented, applying a selection intensity (i) of 1.46 for the strains with lower ethanol yield; therefore, a selection response of 5.8% in one generation was expected (Table 1). From these crosses, more than 900 potential hybrids were generated through micromanipulation (Table S2). We then used between one and four of the available SSRs for each cross to confirm the true hybrid nature of most of these strains, allowing us to confirm 162 of the 632 (25.6%) potential hybrids evaluated, all of which were phenotyped (Tables S2 and S3).

**Figure 1:**
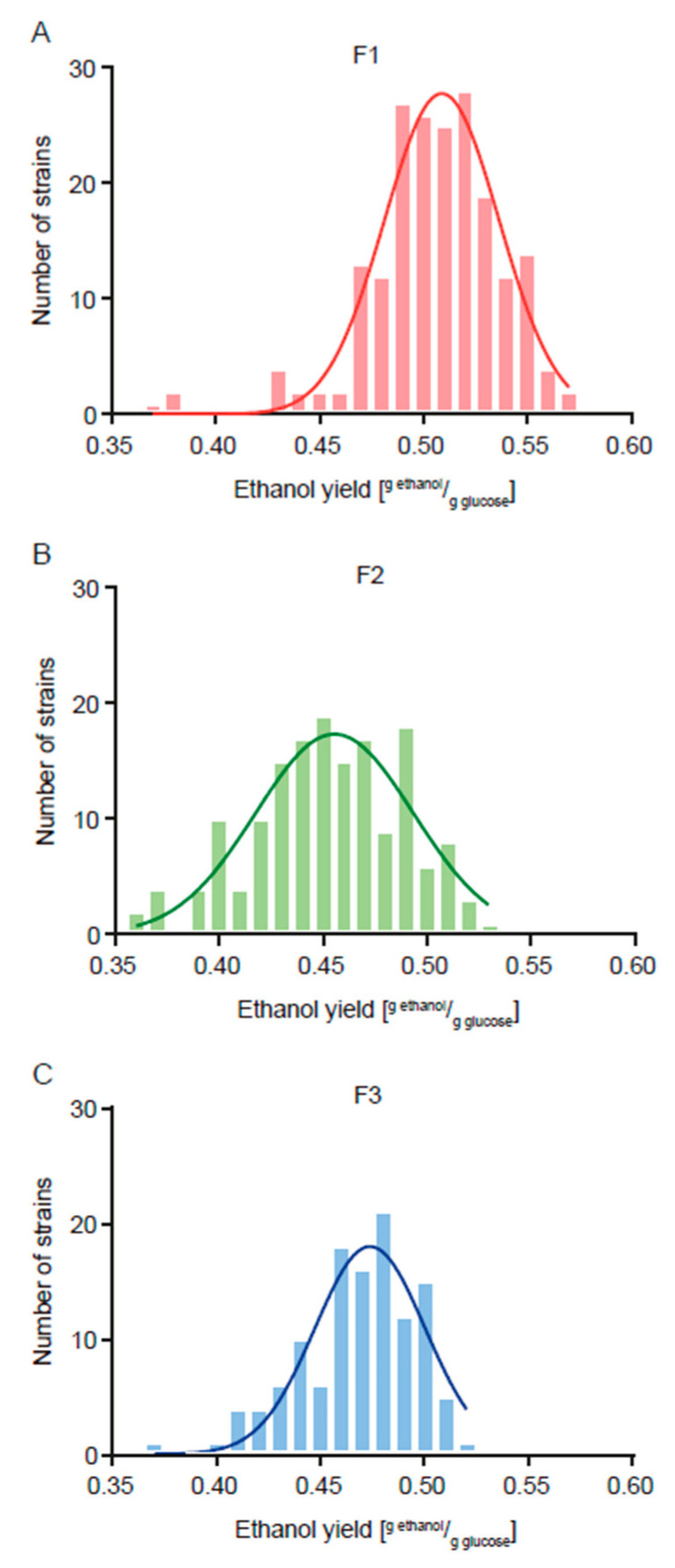
Frequency histograms for the phenotypic values of ethanol yield. Phenotypic values of (**A**) the F1 population, (**B**) F2 population and (**C**) F3 population are shown. For each case, the Gaussian non-linear regression is shown.

**Figure 2:**
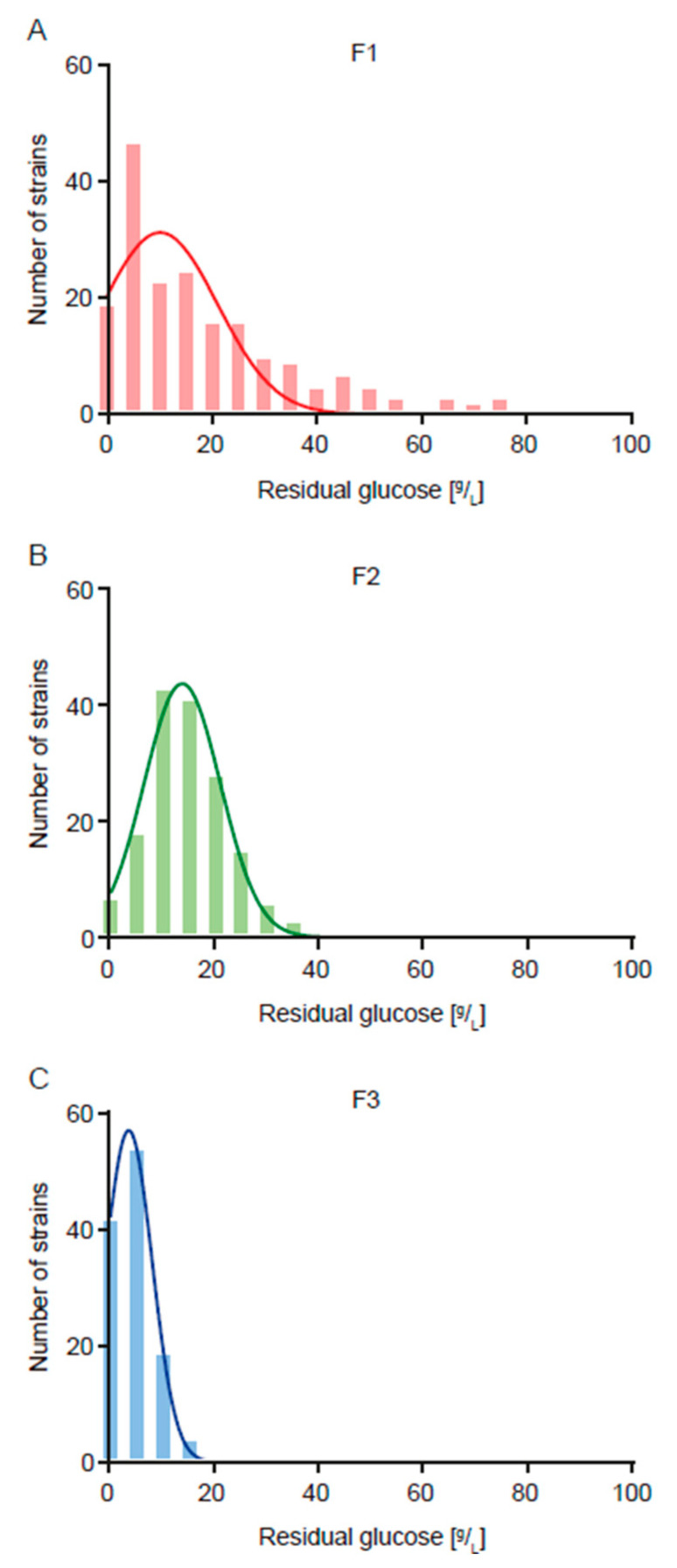
Frequency histograms for the phenotypic values of residual glucose. Phenotypic values of (**A**) the F1 population, (**B**) F2 population and (**C**) F3 population are shown. For each case, the Gaussian non-linear regression is shown.

The F2 population showed a mean value for the trait of 0.452 g ethanol/g sugar, which represents a decrease of 10.7% compared to that of the F1 population (Figure 1, Table 2 and Figure S1). However, even though this decrease in ethanol yield caused a concomitant decrease in residual glucose from 19.7 g/L to 14.6 g/L (25.9%) (Figure 2, Table 2 and Figure S1), this last value is still very high in terms of industrial applications (where values lower than 5.0 g/L are expected). Because of this, we carried out a second round of breeding aimed at reducing residual glucose. The F2 population showed a mean value for this trait of 14.6 g/L, with a minimum of 0.2 g/L and a maximum of 40.0 g/L, and a heritability value of 0.944 (Table 1 and Table 2, and Figure S1).

In total, 17 strains (10.5%) of the F2 population, belonging to 8 families, were selected as parental strains to produce a third filial generation (F3 population) composed of 12 families, applying a selection intensity (i) of 1.73 for the strains with lower residual glucose; therefore, a selection response of 86.4% was expected (Table 2). From these crosses, 344 potential hybrids were generated through micromanipulation, and 132 (38.4%) of them were confirmed as true hybrids; 120 of these hybrid strains were then phenotyped (Tables S2 and S4).

The F3 population showed a mean value for the residual glucose trait of 4.3 g/L, which represents a decrease of 70.6% compared to that of the F2 population (and 78.2% compared to the F1 population) (Figure 2, Table 2 and Figure S1). While the F3 population presented a slight increase (3.5%) in terms of ethanol yield compared to the F2 population, this value still is 9.2% lower than that of the F1 population (Figure 1, Table 2 and Figure S1).

It is important to note that the F1 population also showed a high residual fructose concentration (53.9 g/L) (Table S1), which was reduced by the two rounds of breeding (46.9 g/L and 29.1 g/L for F2 and F3, respectively) (Tables S3 and S4 and Figure S2). However, these residual fructose values are still higher than expected, although the high values also obtained for the commercial strain EC1118 (17.6 g/L) indicate that they may have resulted from our experimental setup, consisting of a high initial sugar level (250 g/L), a small volume (12 mL) and a lack of agitation.

### 3.2. Improved Strains with Applied Potential for the Wine Industry

Once the two rounds of breeding were carried out, we were able to identify 17 strains belonging to the F3 population that stood out for their low ethanol yields and low residual glucose values when carrying out fermentations in synthetic must at the laboratory level (Table 3). This is particularly evident when comparing their fermentative behaviors with those of strain EC1118, both a commercial strain commonly used in the wine industry and a control strain commonly used in scientific work studying wine yeasts, which showed higher values for both traits (Table 3).

To continue the search for the strains with the greatest potential for industrial application, we looked for those that were also resistant to the SO_2_ commonly used in the wine industry, this being an important characteristic to ensure their industrial applicability. To achieve this, the 17 strains belonging to the F3 population previously selected based on their ability to correctly complete alcoholic fermentation (in terms of residual glucose content) with low performance in the conversion of sugar into ethanol were grown in synthetic must in a microculture to evaluate their resistance to a wide range of different SO_2_ concentrations (20, 40, 60, 80 and 100 ppm).

From this group of strains, six were selected (C2-1B4, C7-1B7, C7-2B2, C7-2C2, C7-3A10, and C10-2I5) since they presented the highest levels of resistance to this substance (Table S5). We took into especial consideration their resistance to 40 and 60 ppm of SO_2_, as these are concentrations close to the maximum permitted by different legislations (although some countries may have higher ones) (Table 3). Notably, some of these strains showed resistance levels comparable to or even higher than those of the EC1118 strain.

### 3.3. Improved Strains Able to Produce Wines with Commercial Potential

The six previously selected strains (C2-1B4, C7-1B7, C7-2B2, C7-2C2, C7-3A10, and C10-2I5) were used to produce wine on a pilot scale under winery conditions, using natural musts of Sauvignon blanc grapes (white wine) and Carmenere grapes (red wine). None of the improved strains showed implantation problems in either of the two musts used (Table 4), which is indicative that these strains can grow without problems in both types of must, a necessary condition for the fermentation process to be carried out. Furthermore, all six improved strains managed to effectively complete the fermentation process, considering the low concentration of reducing sugars and the alcohol content of the wines produced (Table 5).

In terms of ethanol yield, the Sauvignon blanc must presented an initial sugar concentration of 22.8 °Bx with an estimated potential alcohol value of 13.3% ABV, while the Carmenere must presented an initial sugar concentration of 24.4 °Bx with an estimated potential alcohol value of 14.4% ABV. One of the improved strains (C7-1B7) had a lower alcoholic degree (0.3% ABV less) than expected in Sauvignon blanc must, while all the improved strains had a lower alcoholic degree than expected in Carmenere must, highlighting strain C2-1B4 (1.5% ABV less) (Table 5). These two strains (C2-1B4 and C7-1B7) were also shown to be genetically stable through a comparison of mtDNA RFLP banding patterns and electrophoretic karyotyping (Figure S3), and thus were ultimately selected for intellectual property protection. This shows the ability of these strains to carry out the alcoholic fermentation process with a lower ethanol yield.

**Figure 3:**
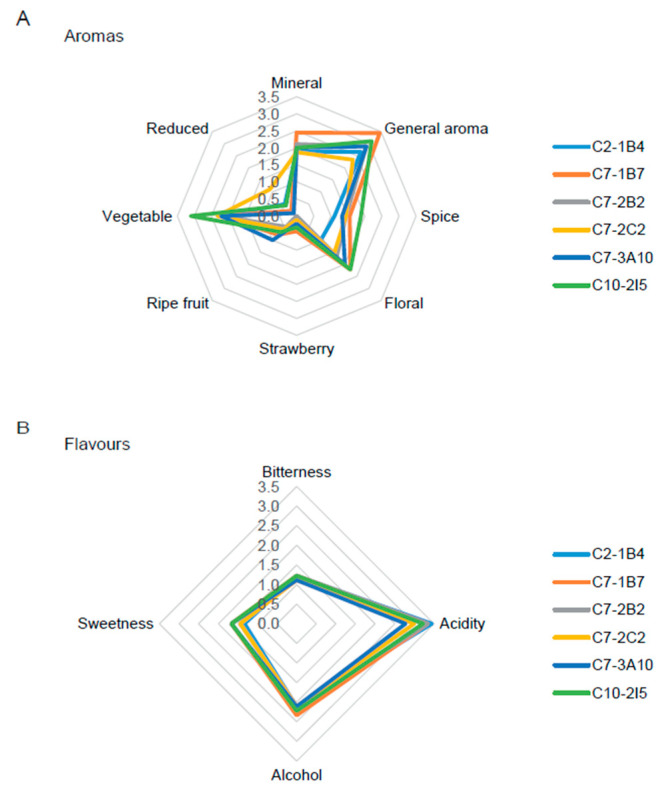
Radar charts for sensory analyses of white wines. Descriptive attributes for (**A**) aromas and (**B**) flavors are shown for each improved strain.

**Figure 4:**
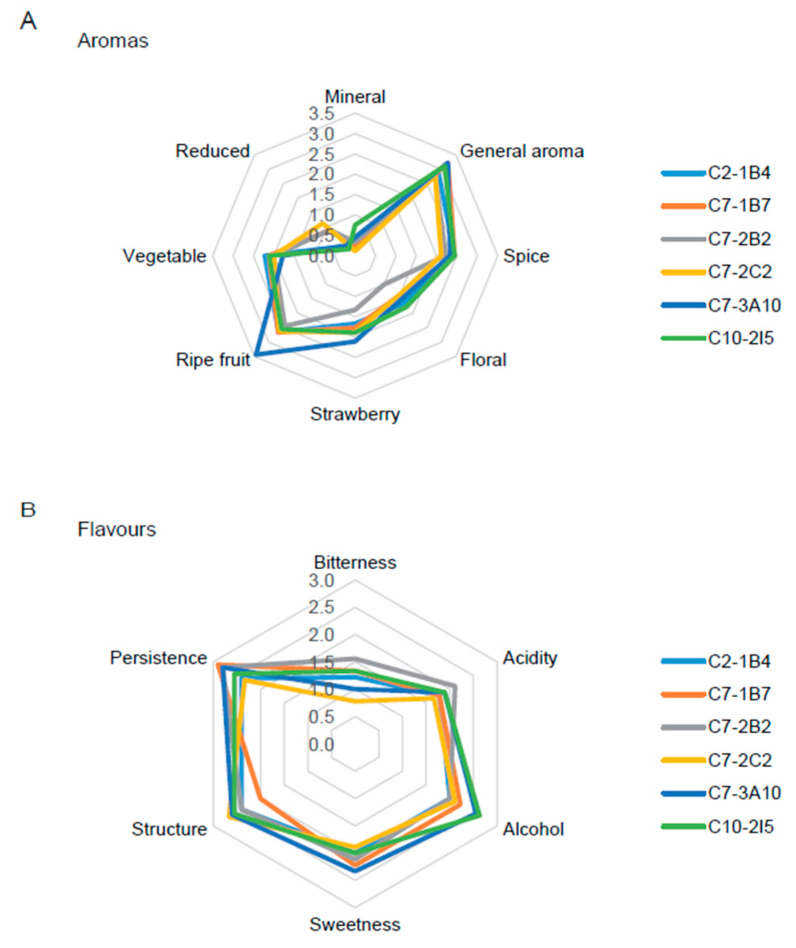
Radar charts for sensory analyses of red wines. Descriptive attributes for (**A**) aromas and (**B**) flavors are shown for each improved strain.

Finally, an independent panel of expert judges (all of them oenologists) carried out a sensory analysis of the wines produced with the improved strains through affective and descriptive sensory tests. The results show that there are no statistically significant differences at the sensory level, neither at the affective level (Table 6) nor at the descriptive level (Figure 3 and Figure 4 and Table S6), between the wines produced by the different improved strains. Both the white and red wines produced by the improved strains obtained good scores in affective terms, being evaluated to have between 3 (“I am indifferent”) and 4 (“I like it”) points on average, except for the white wine produced by the strain C7-1B7, which was evaluated between 4 (“I like it”) and 5 (“I like it a lot”) points (Table 6).

On the other hand, both white and red wines produced by the improved strains stood out for their general aroma, with more vegetal tones in the white wines and ripe fruit tones in the red wines, while in terms of flavor, both were characterized by their low bitterness, highlighting white wines for their acidity and red wines for their balance in terms of alcohol, sweetness, structure, and persistence (Figure 3 and Figure 4 and Table S1). Taken together, these results are indicative that these strains can produce wines that may be liked by a considerable portion of consumers and, therefore, that may be potentially marketable.

## 4. Discussion

High alcohol levels in wine due to high concentrations of sugar in the grapes at harvest time represent one of the main, current problems in the wine industry, with the average alcohol concentration in wines increasing by an average of 2% ABV in the last 30 years and resulting in unbalanced or “flabby” wines [5,6]. In the present work, we carried out a two-round breeding program to obtain improved yeast strains with lower ethanol yield, thus generating a potential microbiological solution to this industrial problem. To achieve this, the first round of breeding was aimed at reducing ethanol yield specifically; we selected ethanol yield and not ethanol production as a target phenotype, because strains could potentially only have lower ethanol production due to them consuming less sugar, and a strain with commercial potential should be able to consume nearly all the sugars present in the grape must.

Regarding ethanol yield, high heritability (0.619) was obtained, where much of the observed variation was due to the additive effects of the genes, making this group of strains have high selection potential (Table 1). However, there is still a high percentage of unexplained residual variation, which can be avoided in future designs by including variables such as time, the position of the family in the physical space, and/or of the professional in charge of the crossing (in the case where more than one person is in charge), to obtain more robust heritability estimates, eliminating artifacts in the design. In any case, this heritability value is in line with those that have been previously obtained for ethanol production, which are between 0.75 and 0.80 [13,19,20]. More importantly, this round of breeding was very successful in reducing ethanol yield on a population scale, with a 10.7% reduction (Figure 1, Table 2 and Figure S1).

In addition to reducing ethanol yield, this first round of breeding also caused a reduction in residual glucose (25.9%) (Figure 2, Table 2 and Figure S1). In previous work, we saw high phenotypic and genetic correlations between ethanol production and residual glucose (and fructose) [13,19,20], which can also be seen here in terms of the significant phenotypic (0.2) and genetic (0.73) correlations between ethanol yield and residual glucose. However, the residual glucose values were still very high in terms of industrial application (where values lower than 5.0 g/L are expected), so we decided to carry out a second round of breeding aimed at reducing this phenotype. We again obtained a high heritability value (0.944), even higher than the previously obtained one (0.728) [13,19,20], and successfully reduced residual glucose by 70.6% on a population scale (Figure 2, Table 2 and Figure S1), obtaining several strains with values compatible with industrial application (Table 3). Together, these results highlight the great potential of yeast breeding programs, indicating the possibility to carry out other breeding programs with commercial potential for closely related phenotypes, such as high glycerol production (an important metabolite for the wine body) and high ethanol production (for bulk wine sales).

This breeding program can not only be a tool to generate novel yeast strains with industrial applications, but can also be an opportunity to study the genetic basis underlying the traits of the selected strains and identify which differences in key genes and/or gene networks explain the phenotypic differences for ethanol yield. As well as other oenological traits, ethanol production and residual sugar are complex traits that are determined by multiple quantitative trait loci (QTL) [50]. Several genes have been linked to the natural variation in the ability of yeast cells to perform wine fermentation and produce ethanol, for example by QTL mapping [51,52]. In the context of the present work, an interesting approach would be to study the genetic determinants that have been inherited from the F0 to F3 population, by sequencing improved strains (F3) and their parents (F2), grandparents (F1) and great-grandparents (F0), to gain insights into the genetic variants underlying the reduction in ethanol yield and residual glucose. This could also be compared against the results of a future breeding program aimed at increasing ethanol yield, which may lead to a better understanding of fermentative metabolism under winemaking conditions and its corresponding genetic basis. It will be interesting to analyze whether such an analysis confirms the involvement of only known genes (such as *PDC1* and *ADH1*) and metabolic pathways (such as glycolysis), or whether other genes previously unrelated to alcoholic fermentation can also be found to be involved.

Interestingly, the F1 population showed not only a high mean population value of residual glucose (19.7 g/L) but also an even higher value for residual fructose (53.9 g/L) (Table S1). The two rounds of breeding reduced this value in both the F2 (46.9 g/L) and F3 (29.1 g/L) populations (Tables S3 and S4 and Figure S2), which is consistent with the previously high phenotypic and genetic correlations previously observed between residual glucose and residual fructose [13]. However, these residual fructose values were still higher than expected and could have been reduced further if the second breeding round had been aimed at reducing total residual sugar (rather than just residual glucose), or if we had carried out a third breeding round aimed at reducing residual fructose. However, it is important to note that the commercial strain EC1118 also showed a high level of residual fructose (17.6 g/L), in contrast with its low level of residual glucose (3.9 g/L) (Table S4), which may indicate that these high levels of residual fructose in both the F3 and control strains were due to our experimental setup, consisting of small-volume (12 mL) fermentations without agitation. This can be more obviously seen when comparing these results with those obtained in the natural must (10 L fermentations), in which none of the improved strains had total residual sugar values greater than 3.0 g/L (Table 5). This highlights the need to use larger laboratory-scale fermentation volumes to obtain more reliable results (ideally up to 200 mL).

As a result of this two-round breeding program, we selected six strains (C2-1B4, C7-1B7, C7-2B2, C7-2C2, C7-3A10 and C10-2I5) with potential industrial application, in terms of their low ethanol yield, low residual glucose and high SO_2_ resistance on a laboratory scale (Table 3); SO_2_ is an antimicrobial substance commonly used by the wine industry to prevent the growth of contaminating microorganisms [53]. We then produced white (Sauvignon blanc) and red (Carmenere) wines on a pilot scale using these six improved strains; all of them were shown to be capable of having industrial applications for the wine industry, in terms of implantation (Table 4), residual reducing sugars (Table 5), and organoleptic properties evaluated by a trained panel (Figure 3 and Figure 4, Table 6, and Table S1).

In terms of ethanol production, we compared the results obtained with the improved strains against an estimated potential alcohol value for each grape must. To estimate the potential alcohol level in a wine, winemakers use a value that represents how many grams of sugars (g/L in grape must) are needed to generate an alcoholic degree (% ABV). However, different winemakers could use different values, ranging from 16.7 to 18.0, and even the same winemaker could use different values for white and red wines [54,55]. Furthermore, this value not only depends on the grape variety but also on the yeast strain used [54]. The official European Union (EU) conversion ratio is 1% ABV for every 16.83 g/L, but a correction to 17.0 has recently been proposed [56]; this is the value used by the oenologist who supervised the present study. Using this value, we obtained potential alcohol values of 13.3% ABV and 14.4% ABV for Sauvignon blanc and Carmenere, respectively.

Of these six improved strains, one of them (C7-1B7) had a lower alcoholic degree (0.3% ABV less) than expected in Sauvignon blanc must, while all of them had a lower alcoholic degree than expected in Carmenere must (highlighting strain , with 1.5% ABV less) (Table 5). These strains showed ethanol yield values similar to those of the commercial strain IONYSwf™ (Lallemand), obtained through adaptive evolution; this strain showed an ethanol yield of 0.423 (g ethanol/g sugar) in Shiraz must [6], while strain C7-1B7 had a value of 0.429 (g ethanol/g sugar) in Sauvignon blanc must and strain C2-1B4 had a value of 0.426 (g ethanol/g sugar) in Carmenere must (Table S4).

The different results obtained between these two grape musts may be due to the differences in the initial sugar concentration: The Sauvignon blanc must did not have a particularly high initial sugar concentration (22.8 °Bx), unlike the Carmenere must (24.4 °Bx). These results point towards the potential effect of the initial sugar concentration on ethanol yield, which is an example of a gene–environment interaction, an important phenomenon that impacts breeding programs, as has been previously evidenced [57,58]. However, it is difficult to determine if the differences obtained using these two natural musts were actually due to differences in initial sugar concentration or due to other differences (e.g., fermentation conditions or other specific differences in grape must composition). To determine this, future work needs to evaluate the wines produced by these strains from musts of other grape varieties and/or with different initial sugar concentrations, in order to fully understand the relationship between initial sugar concentration and ethanol production.

Beyond that, the results show that C2-1B4 and C7-1B7 are the strains that have the greatest potential for industrial applications. There are two main future industrial applications: (i) the production of high-quality wines with adequate alcohol content from grape musts with high initial sugar concentrations, and (ii) the production of novel products, such as wines with reduced alcohol content (around 8.0% ABV) marketed to young consumers looking for alternatives to other alcoholic beverages (such as beer). As previously mentioned, future work will focus on the fermentation of other grape must varieties (with or without different initial sugar concentrations), in order to fully understand the industrial potential of these strains. Additionally, it will be important to consider and evaluate other important industrial production conditions, such as fermentation conditions (temperature, nitrogen supplementation, etc.), specific grape must compositions and microbial contamination, that may affect the fermentation performance and stability of the strains. Finally, scaling up production is important, to move from pilot-scale volumes in the laboratory (in the order of 10 L) to real production volumes (in the order of 1000 L).

Finally, it is interesting to note how one of the F3 families (family C7) showed a general tendency to contain strains with lower ethanol yields and residual glucose levels, as well as high levels of SO_2_ resistance. Of the 17 strains selected for SO_2_ resistance evaluation (due to their low levels of ethanol yield and residual glucose), 8 of them are strains belonging to the C7 family (Table 3). Moreover, four of the six improved strains selected for pilot-scale wine production also belong to this family (Table 4); originally, we selected five of the six, but we finally selected strain C2-1B4 over strain C7-1B5 in order to increase the genetic diversity of the selected improved strains in terms of their families of origin (at least three families were represented). An interesting approach could be to study the genetic determinants that have been inherited from F0 to F3 population for the C7 family in comparison with other families, to gain information about the genetic variants underlying the reduction in ethanol yield.

## 5. Conclusions

In conclusion, we were able to obtain non-transgenic genetically improved strains with lower ethanol yields through a two-round breeding program. Six of these strains, which showed industrial potential, were used to produce wine from natural grape musts on a pilot scale, highlighting improved strains “C2-1B4” and “C7-1B7” as those that showed the best results. This opens the door to conducting other breeding programs for closely related phenotypes, such as high glycerol and ethanol production. Furthermore, more studies are required to understand the connection between initial sugar concentration and ethanol yield, as well as the genetic variants underlying this phenotype.

## 6. Patents

CL 202400794: “Cepas de levadura *Saccharomyces cerevisiae* no transgénicas con menor eficiencia en la producción de alcohol” (“Non-transgenic *Saccharomyces cerevisiae* yeast strains with reduced efficiency in alcohol production”).

## Figures and Tables

**Table 1:** Breeding values for the traits under improvement.

	First Round of Breeding (Ethanol Yield)	Second Round of Breeding (Residual Glucose)
Phenotypic variance (V_P_)	0.00107 ± 0.00013	59.691 ± 11.055
Additive genetic variance (V_A_)	0.00066 ± 0.00024	56.345 ± 21.304
Residual variance (V_R_)	0.00041 ± 0.00016	3.346 ± 11.544
Heritability (h^2^)	0.619 ± 0.173 *	0.944 ± 0.202 *
Expected response to selection (G)	0.030	12.62
Percentage of response to selection (%G)	5.8%	86.4%

Statistical analyses of heritability correspond to the log-likelihood ratio test (log-LR test) (*: *p* < 0.05).

**Table 2:** Mean population values for the traits under improvement.

Population	Ethanol Yield ± SD (g Ethanol/g Sugar)	Residual Glucose ± SD (g/L)
F1	0.506 ± 0.032 ^a^	19.7 ± 18.4 ^a^
F2	0.452 ± 0.036 ^c^	14.6 ± 7.7 ^b^
F3	0.468 ± 0.028 ^b^	4.3 ± 3.9 ^c^

SD: Standard deviation. Statistical analyses correspond to ordinary one-way ANOVA using Holm–Šídák’s multiple comparisons tests, with values that have a statistically significant difference (*p* < 0.05) having different superscript letters (a, b and c).

**Table 3:** Phenotypic values of selected improved strains.

Strain	Ethanol Yield (g Ethanol/g Sugar)	Residual Glucose (g/L)	Relative SO_2_ Resistance	
			40 ppm	60 ppm
C1-1C5	0.458	0.9	11%	3%
C2-1B4	0.421	0.9	56%	17%
C3-1B7	0.452	0.7	33%	5%
C3-2A10	0.457	0.2	34%	3%
C6-1B3	0.400	0.6	15%	1%
C6-2C6	0.460	1.3	17%	8%
C7-1B5	0.419	0.4	34%	24%
C7-1B7	0.369	2.0	56%	32%
C7-2A2	0.411	0.1	42%	2%
C7-2B2	0.438	0.0	70%	34%
C7-2C2	0.438	0.0	32%	27%
C7-3A10	0.429	0.1	75%	56%
C7-3A6	0.469	0.8	33%	4%
C7-3B4	0.447	0.0	42%	18%
C10-2I5	0.462	2.5	59%	80%
C12-1A5	0.432	0.8	18%	11%
C12-1A9	0.461	0.2	1%	0%
EC1118	0.482	3.9	69%	56%

**Table 4:** Implantation of selected improved strains in natural grape musts.

Strain	Implantation Percentage	
	Sauvignon Blanc	Carmenere
C2-1B4	90.9%	81.8%
C7-1B7	100%	100%
C7-2B2	100%	90.9%
C7-2C2	100%	100%
C7-3A10	100%	100%
C10-2I5	100%	100%

**Table 5:** Chemical analysis of the wines produced with the selected improved strains.

Strain	Sauvignon Blanc		Carmenere	
	Reducing Sugars (g/L)	Alcohol (% ABV)	Reducing Sugars (g/L)	Alcohol (% ABV)
C2-1B4	0.96	13.6	2.89	12.9
C7-1B7	1.00	13.0	2.77	13.6
C7-2B2	1.08	13.3	2.74	13.7
C7-2C2	1.77	13.4	2.92	13.5
C7-3A10	1.00	13.5	2.98	13.5
C10-2I5	0.82	13.4	2.91	13.4

**Table 6:** Results by strain of the affective test of the wines produced with the selected improved strains.

Strain	Sauvignon Blanc	Carmenere
C2-1B4	3.3 ± 0.9	3.7 ± 1.0
C7-1B7	4.2 ± 0.7	3.0 ± 1.1
C7-2B2	3.9 ± 0.6	3.6 ± 1.1
C7-2C2	3.4 ± 1.1	3.1 ± 1.1
C7-3A10	3.6 ± 0.9	3.9 ± 0.6
C10-2I5	3.2 ± 1.1	3.7 ± 0.9

All values correspond to the mean value ± their standard deviation (SD). Statistical analysis for each must consisted of independent ANOVA tests; for both wines (white and red), *p* values of the F ratio were greater than 0.05, so no statistically significant differences were observed between the strains.

## Data Availability

The original contributions presented in this study are included in the article/Supplementary Materials. Further inquiries can be directed to the corresponding author.

## Supplementary Materials

The following supporting information can be downloaded at https://www.mdpi.com/article/10.3390/jof11020137/s1: Figure S1: Box plots for the phenotypic values of the traits under improvement; Figure S2: Frequency histograms for the phenotypic values of residual fructose; Figure S3. Genetic stability of selected strains; Table S1: Oenological parameters of F1 population; Table S2: Hybris obtained by cross; Table S3: Oenological parameters of F2 population; Table S4: Oenological parameters of F3 population and control strain (EC1118); Table S5: Relative SO_2_ resistance values of selected improved strains; Table S6: Results by attribute of the descriptive test of the wines produced with the selected improved strains.
