# Supplementary material for: Genetically Improved Yeast Strains with Lower Ethanol Yield for the Wine Industry Generated Through a Two-Round Breeding Program
Source: J Fungi (Basel). 2025 Feb 11;11(2):137. doi: 10.3390/jof11020137 (PMC11855951; doi:10.3390/jof11020137)
Supplement: Supplementary file 1 [file jof-11-00137-s001.zip › Figure_S1.pdf]

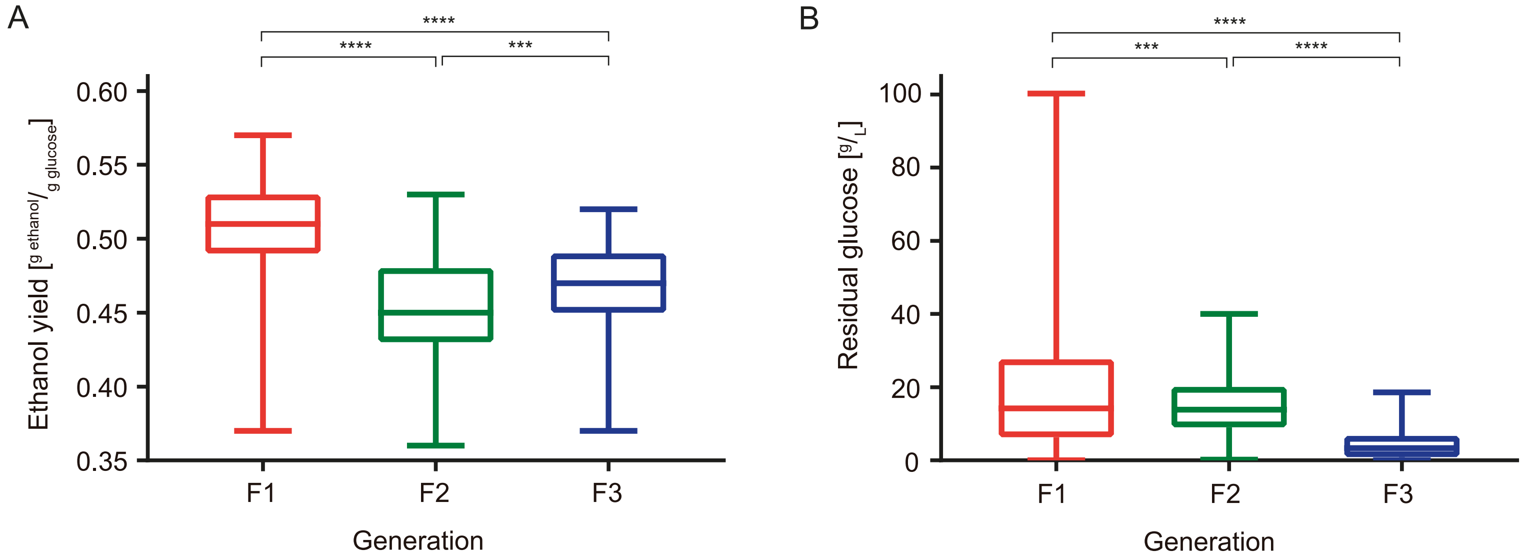

Figure S1. Box plots for the phenotypic values of the traits under improvement. Phenotypic values of (A) ethanol yield and (B) residual glucose are shown. In each case, the vertical line goes from the minimum to the maximum phenotypic value obtained. Statistical analyses correspond to ordinary one-way ANOVA using Holm-Šídák's multiple comparisons tests (****: $p<0.0001$, ***: $p<0.001$).
