# Supplementary material for: Genetically Improved Yeast Strains with Lower Ethanol Yield for the Wine Industry Generated Through a Two-Round Breeding Program
Source: J Fungi (Basel). 2025 Feb 11;11(2):137. doi: 10.3390/jof11020137 (PMC11855951; doi:10.3390/jof11020137)
Supplement: Supplementary file 1 [file jof-11-00137-s001.zip › Figure_S2.pdf]

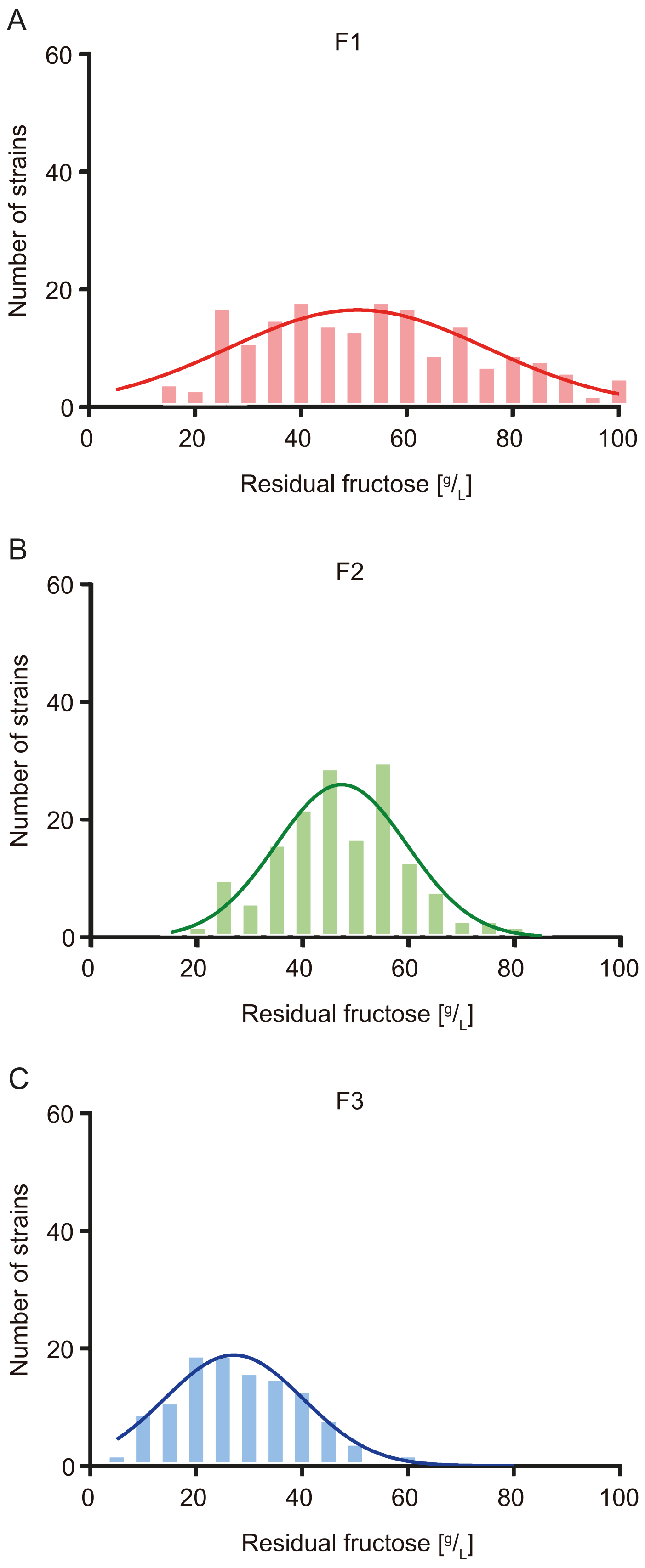

Figure S2. Frequency histograms for the phenotypic values of residual fructose. Phenotypic values of (A) F1 population, (B) F2 population and (C) F3 population are shown. In each case, the Gaussian non-linear regression is shown.
