# Supplementary material for: Genetically Improved Yeast Strains with Lower Ethanol Yield for the Wine Industry Generated Through a Two-Round Breeding Program
Source: J Fungi (Basel). 2025 Feb 11;11(2):137. doi: 10.3390/jof11020137 (PMC11855951; doi:10.3390/jof11020137)
Supplement: Supplementary file 1 [file jof-11-00137-s001.zip › Figure_S3.pdf]

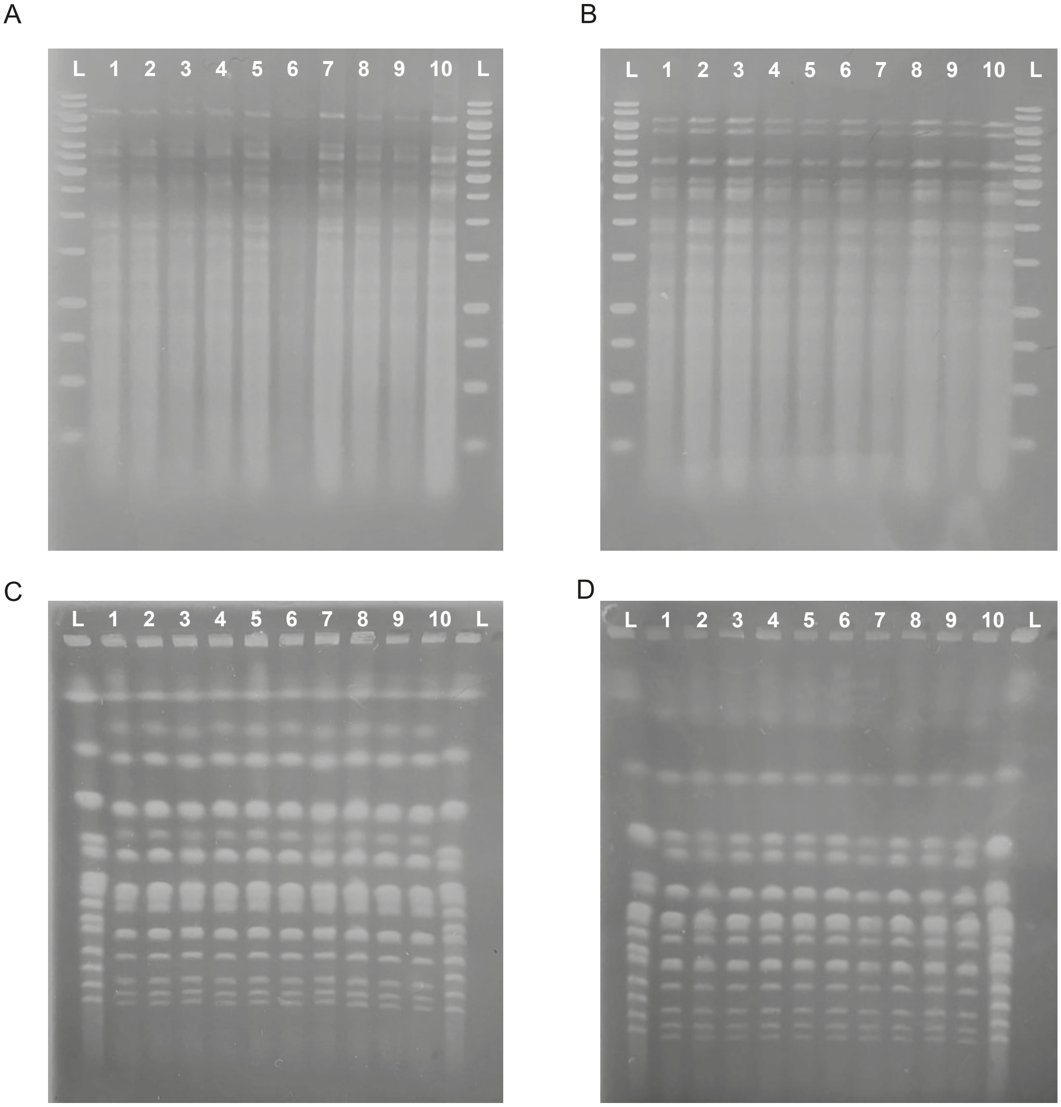

Figure S3. Genetic stability of selected strains. Genetic stability of strains (A-C) C2-1B4 and (B-D) C7-1B7 was evaluated by (A-B) mtDNA RFLP banding patterns and (C-D) electrophoretic karyotyping of strains. In all cases, lanes 1 to 10 correspond to DNA samples extracted from 10 different colonies for each strain, while lane 'L' correspond to de DNA ladder used in each case: (A-B) GeneRuler 1 kb Plus DNA Ladder (Thermo Fisher Scientific, MA, USA) and (C-D) CHEF DNA Size Marker, 0.2–2.2 Mb, S. cerevisiae Ladder #1703605 (Bio-Rad, CA, USA).
